# Role of Optic Nerve Sheath Diameter on Mortality Prediction in Patients Admitted to the Intensive Care Unit from the Emergency Department

**DOI:** 10.3390/diagnostics15040490

**Published:** 2025-02-17

**Authors:** Kazım Ersin Altınsoy, Bahar Uslu Bayhan

**Affiliations:** 1 Department of Emergency Medicine, Gaziantep Islam Science and Technology University, 27470 Gaziantep, Türkiye; 2 Department of Anesthesia and Reanimation, Gaziantep City Hospital, 27470 Gaziantep, Türkiye; dr.b.uslu@gmail.com

**Keywords:** cerebrovascular events, emergency department, intensive care unit, magnetic resonance imaging, mortality prediction, optic nerve sheath diameter

## Abstract

**Background/Objectives**: Cerebrovascular events (CVEs) are a leading cause of intensive care unit (ICU) admissions from the emergency department, often associated with high morbidity and mortality rates. Identifying reliable, non-invasive predictors of mortality in these patients is critical for improving prognostic accuracy and guiding therapeutic strategies. This retrospective cohort study evaluates the predictive value of the optic nerve sheath diameter (ONSD), measured using magnetic resonance imaging (MRI), in determining mortality among ICU patients with CVEs. **Methods**: This single-center, retrospective observational study included 102 patients diagnosed with CVEs and admitted to the ICU at Gaziantep City Hospital between October 2023 and March 2024. This study adhered to the Declaration of Helsinki. Ethics approval was obtained from Gaziantep Islam Science and Technology University (Decision No. 394.36.08), and the requirement for informed consent was waived due to the retrospective design. The sample size was determined using G-Power 3.1.9.4. **Results**: A statistically significant positive correlation was observed between the ONSD and mortality (*p* = 0.002). Patients with higher ONSD values demonstrated an increased mortality risk, underscoring the potential prognostic value of ONSD measurements in this population. **Conclusions**: MRI-based ONSD measurement offers a non-invasive method for predicting mortality in ICU patients with CVEs. Its integration into routine diagnostic protocols could enhance clinical decision-making and patient outcomes. Further multicenter studies are warranted to validate these findings and standardize ONSD measurement techniques.

## 1. Introduction

Intensive care units (ICUs) have the highest mortality rates among hospital wards. Efforts to reduce mortality in ICUs have remained a significant topic of research interest for investigators in every era. The most common neurological event necessitating admission to the intensive care unit is cerebrovascular events (CVEs). Therefore, evaluating mortality in cerebrovascular diseases is of primary importance [1]. Cerebrovascular events are predominantly ischemic but sometimes hemorrhagic and often lead to permanent neurological dysfunction [1,2].

Intracranial pressure (ICP) plays a critical role in determining the prognosis in neurological conditions, including CVEs. Elevated ICP can lead to cerebral ischemia, impaired cerebral metabolism, and neurological deterioration, all of which are associated with increased mortality [3,4]. The major consequences of increased intracranial pressure are recurrent cerebral ischemic attacks, changes in cerebral blood flow, impairment of cerebral metabolism, and losses in cerebral sensory transmission [4]. Increased intracranial pressure, especially in cases unresponsive to treatment, is associated with poor outcomes in traumatic brain injury [5]. The continuity of the optic nerve sheath with the meninges and subarachnoid space in the intracranial area has enabled its use as an indicator of intracranial pressure. Studies have proved that the measurement of the optic nerve sheath diameter using ultrasound or magnetic resonance imaging (MRI) techniques serves as a non-invasive indicator of increased intracranial pressure [6]. The current use of magnetic resonance imaging as a neurodiagnostic tool in cerebrovascular diseases has validated its suitability for measuring the optic nerve sheath diameter in all patients [7]. This method, which is advantageous due to its non-invasive nature and broad applicability, demonstrates a strong correlation with invasive measurements, as reported in the literature [8]. Consequently, it helps mitigate complications such as infection and bleeding associated with invasive diagnostic procedures, including lumbar puncture and ventriculostomy [9].

Our study assumes that measuring the optic nerve sheath diameter using magnetic resonance imaging in intensive care unit patients diagnosed with cerebrovascular events is an effective method for predicting mortality.

## 2. Materials and Methods

This retrospective, observational cohort study was conducted between October 2023 and March 2024 at Gaziantep City Hospital Emergency Service. Patients presenting with suspected acute stroke, diagnosed with cerebrovascular events (CVE) based on imaging findings and expert consultation, and subsequently admitted to the intensive care unit (ICU) for follow-up and treatment were included. The single-center design ensures consistency in data collection, imaging protocols, and patient management, reducing variability in measurement techniques and clinical assessments. This study received ethics committee approval from Gaziantep Islam Science and Technology University (Meeting No. 36, 26 March 2024; Decision No. 394.36.08). Using G-Power version 3.1.9.4 (Kiel University, Kiel, Germany) to calculate the sample size, 102 patients were enrolled. Due to the retrospective nature of this study, the requirement for informed consent was waived in accordance with local regulations. This study adhered to the principles of the 2008 Declaration of Helsinki. In our emergency department, cranial imaging is performed early in patients suspected of having a cerebrovascular event (CVE) in accordance with current guidelines. To ensure homogeneity in our study, we included only patients for whom the time interval between stroke onset and ONSD measurement was within 1 h. Patients who underwent magnetic resonance imaging (MRI), with or without contrast, for diagnostic purposes were included.

Inclusion criteria were as follows:

Age ≥ 18 years.

Patients who first applied to our hospital’s emergency department and were later admitted to our intensive care unit.

Patients who presented with symptoms and findings suspicious for acute stroke and were subsequently diagnosed with CVE using imaging methods and expert consultation in accordance with the guidelines.

Patients who underwent brain magnetic resonance imaging (MRI) with or without contrast as a diagnostic imaging method.

Patients with no known history of previous intracranial pressure (ICP) elevation or conditions associated with chronic ICP elevation.

Absence of any additional intracranial disease.

Patients under endotracheal intubation or being followed with spontaneous respiration.

Exclusion criteria were as follows:

Patients who did not initially present to the emergency department.

Patients who did not undergo magnetic resonance imaging at our hospital or whose magnetic resonance imaging weakly visualized the optic nerve.

Patients with known additional cranial diseases such as aneurysm, hydrocephalus, or intracranial mass.

Patients who left the hospital against medical advice before completion of treatment.

Following the inclusion and exclusion criteria, this study included a total of 102 patients diagnosed with cerebrovascular events.

Patient demographics including age, gender, and laboratory parameters upon initial presentation (Glucose, AST, ALT, ALP, GGT, Albumin, CRP, Lipase, Creatinine, Urea, CK, Na, K, Calcium, Procalcitonin, Phosphorus, Magnesium, WBC, Neutrophil, Lymphocyte, Monocyte, Hematocrit, Platelet, MPV) were recorded. Patient data were retrieved from the hospital information system. Information was recorded for each patient regarding their admission to the intensive care unit (ICU) under either endotracheal intubation or spontaneous respiration, APACHE II score, Glasgow Coma Scale (GCS) score upon initial presentation, expected mortality rate, location of the intracranial event (e.g., right hemisphere, left hemisphere, brainstem, frontal, parietal, temporal, or occipital lobe), type of cerebrovascular event (hemorrhagic or ischemic), duration of ICU stay, discharge disposition (deceased or transferred to another service), presence of additional comorbidities (e.g., lung or cardiac pathology), and optic nerve sheath diameter (ONSD) measurements obtained via magnetic resonance imaging (MRI).

To ensure accurate and consistent measurements, the ONSD was measured by a single physician using MRI scans. Data collection, analysis, and measurement were conducted independently by different researchers.

Neurodiagnostic MRI scans were performed using a 1.5 Tesla MRI machine (Siemens Avanto, Erlangen, Germany). The magnetic resonance images of patients were acquired in the T2-weighted axial plane, ensuring optimal visualization of the eyeballs. The ONSD was measured 3 mm posterior to the eyeball, where the optic nerve sheath appeared most distinct. The distance between the dural sheaths, which appeared hypointense adjacent to the hyperintense subarachnoid space surrounding the optic nerve, was measured. To obtain the most accurate result, each measurement was performed three times per eye, and the averages were recorded as a single measurement for each eye [9,10].

All MRI examinations were performed using a 1.5T MRI system (GE Healthcare, Milwaukee, WI, USA) equipped with a maximum gradient field strength of 22 mT/m and a square head coil. The MRI sequences used in this study are described below: Fluid-attenuated inversion recovery (FLAIR) sequence (TE = 84 ms, TR = 8000 ms, inversion time = 2000 ms, and bandwidth = 20.83 Hz). FLAIR images were acquired in the axial plane with a field of view (FOV) of 240 mm × 240 mm, a matrix size of 256 × 224, and a slice thickness of 5 mm with a 1.5 cm slice gap. Axial T2-weighted MRI scans were conducted using a single-shot spin-echo echo-planar imaging sequence. The sequence covered the entire brain with 20 contiguous slices obtained as two interleaved sequences consisting of four averages, resulting in slices with a thickness of 5.0 mm and a slice gap of 1.5 mm. Other parameters were set as follows: TR = 8000 ms, TE = 88 ms, acquisition matrix size of 96 × 96, FOV = 24 cm, and an in-plane resolution of 2.5 mm.

### Statistical Analysis

G-Power version 3.1.9.4 (University of Kiel, Kiel, Germany) software was used to determine the sample size. ROC analysis was performed to determine the sensitivity and specificity of survival status based on the optic nerve sheath diameter (ONSD). Microsoft EXCEL 2019 (Version 16.0, Washington, DC, USA) was used for ROC analysis. The patients included in this study were evaluated as male, female, and general. Sensitivity and specificity rates for each group were obtained. The Kolmogorov–Smirnov test was used to ensure compliance with normal distribution. Bilateral relationships between variables were investigated with Pearson correlation. In all statistical tests, ALPHA (α) was accepted as 0.05.

Subgroup analyses were not performed in this study. Potential confounding variables such as comorbidities and treatment differences were not adjusted for, which could impact the observed associations.

## 3. Results

This study analyzed 102 patients, evaluating the relationship between optic nerve sheath diameter (ONSD) and mortality, laboratory parameters, and brain pathology. Our findings indicate significant correlations between the ONSD and mortality (*p* = 0.002), expected death rate (*p* = 0.004), and white blood cell (WBC) count (*p* = 0.005).

**Figure 1:**
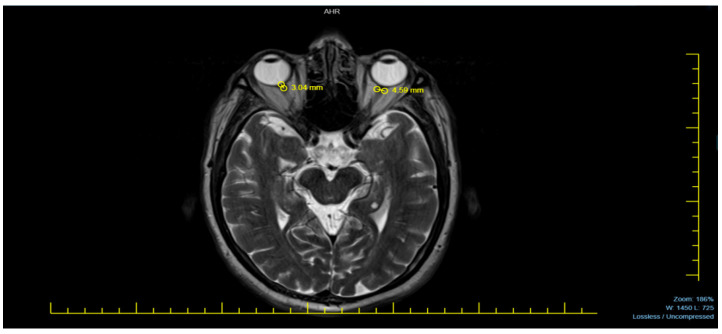
A sample of an MRI.

Figure 1 provides a representative MRI sample used in measuring the ONSD, illustrating the method applied in this study.

Table 1 presents the demographic characteristics of the study population, including gender distribution and age group classification. Out of the 102 patients included in this study, 62 (60.8%) were female and 40 (39.2%) were male. Regarding age distribution, nine patients (8.8%) were between the ages of 18–45, thirty-two patients (31.4%) were aged 46–65, and the majority, sixty-one patients (59.8%), were aged 66 and older.

The Kolmogorov–Smirnov test was used to evaluate the normality of the data for both the optic nerve sheath diameter (ONSD) and APACHE II scores. The results confirmed a normal distribution for the ONSD (*p* = 0.13, D = 0.078, n = 102) and APACHE II scores (*p* = 0.08, D = 0.083, n = 102).

The Spearman rank correlation test revealed a statistically significant positive correlation (r = 0.441) between the optic nerve sheath diameter (ONSD) and mortality/survival status (*p* = 0.002, n = 102). This indicates a 44.1% association, highlighting the relationship between an increased ONSD and higher mortality risk.

The Pearson correlation coefficient analysis revealed a statistically significant positive correlation (r = 0.282, *p* = 0.004, n = 102) between the optic nerve sheath diameter (ONSD) and the expected mortality rate. This result indicates a 28.2% association, suggesting that higher ONSD values are linked to an increased expected mortality rate.

The correlation between the ONSD and the brain region where the event occurred was also examined. The correlation values were close to zero, indicating no significant association (*p* > 0.05), as shown in Table 2. The optic nerve diameter was evaluated based on the pathological region in the brain, and no statistically significant differences were observed when compared.

A statistically significant negative correlation was observed between the optic nerve sheath diameter (ONSD) and hemorrhagic/ischemic events (r = −0.196, *p* = 0.03, n = 102). This finding suggests that as ONSD values increase, the likelihood of hemorrhagic rather than ischemic events decrease by 19.6%.

The correlation between the ONSD and laboratory data was analyzed. For most parameters, no significant correlations were found, as shown in Table 3. However, significant positive correlations were observed between the ONSD and white blood cell count (r = 0.275, *p* = 0.01) and platelet count (r = 0.2, *p* = 0.04), as detailed in Table 4.

An additional analysis was conducted to evaluate the relationship between platelet count and mortality/survival status. The analysis revealed a weak positive correlation (r = 0.129), which was not statistically significant (*p* = 0.195, n = 102). These findings suggest no significant association between platelet count and patient survival outcomes.

Receiver Operating Characteristic (ROC) Analysis

This study analyzed data from 102 patients, comprising 40 males and 62 females. The mean optic nerve sheath diameter (ONSD) was 4.87 mm (SD = 1.03) across the entire population. Gender-specific analyses revealed that the mean ONSD was 4.78 mm (SD = 1.05, *p* = 0.200) for females and 5.01 mm (SD = 0.99, *p* = 0.051) for males. A normal distribution was confirmed for ONSD values in males, females, and the overall cohort (*p* > 0.05).

Based on the available data, ROC analysis was performed for values below one standard deviation from the mean, at the mean, and above one standard deviation from the mean. Table 5 demonstrates that as ONSD increases, sensitivity increases, while specificity decreases. The closest sensitivity and specificity values were observed around the mean ONSD.

**Figure 2:**
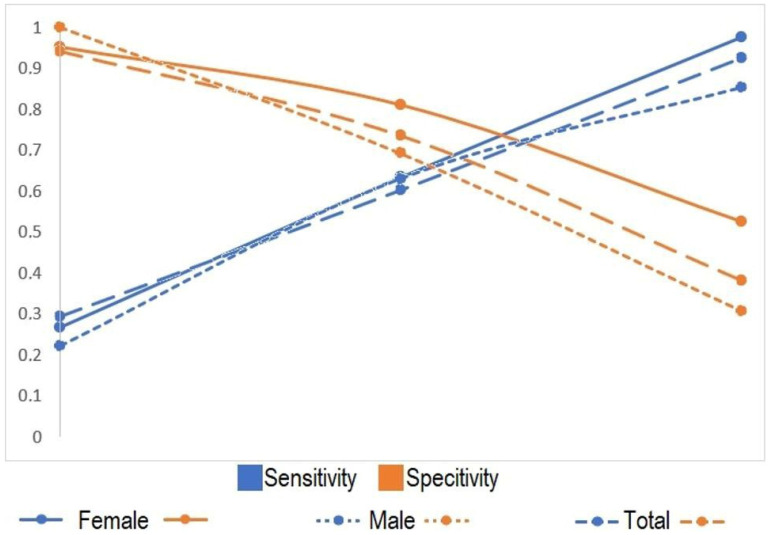
Representation of the change in sensitivity and specificity values for females, males, and overall.

Figure 2 illustrates the variations in sensitivity and specificity values with respect to the ONSD.

## 4. Discussion

Cerebrovascular diseases are defined as the primary pathological condition involving damage to or impairment of blood vessels affecting the brain, which can occur in one or multiple regions of the brain due to ischemia or hemorrhage and can be either permanent or temporary. They rank second among the causes of death in the population aged 60 and above [11]. Intracranial hypertension, which commonly occurs in cerebrovascular events, is indeed a significant cause of mortality [3]. Invasive methods are considered the gold standard for measuring intracranial pressure. However, due to their invasive nature, they cannot be applied in every center. Therefore, there has been a growing trend towards research into alternative methods.

With the increase in intracranial pressure, the meninges stretch, and this is reflected in the optic nerve sheath, which is continuous with the meninges. While optic disc edema has been accepted as an indicator of increased intracranial pressure, it has not been useful in planning treatment or predicting mortality, as it typically appears days later. Instead, it has been observed that an increase in the ONSD can be detected seconds after an increase in intracranial pressure, making it a potentially valuable tool for treatment planning and predicting mortality [10]. Measuring the ONSD is therefore very valuable. Indeed, as many studies in the literature have shown, Geeraerts et al. (2008) demonstrated a correlation between an increased ONSD and high ICP [12]. For this reason, we can use ONSD measurement as a marker in all diagnoses that may cause increased intracranial pressure (for example, head trauma). In our study, we investigated the utility of ONSD measurement in determining mortality in patients admitted to the intensive care unit with a diagnosis of cerebrovascular events. The results we found were promising. We identified a significant correlation between the optic nerve sheath diameter and mortality/survival status (*p* < 0.05).

There are many studies available that measure the optic nerve sheath diameter using magnetic resonance imaging [10,12,13]. In the literature, the MRI-based measurement of intracranial pressure (ICP) has been accepted as a non-invasive measurement method. When conducting a literature review, studies measuring the optic nerve sheath diameter (ONSD) with MRI show variations in imaging techniques and device specifications, resulting in images of different resolutions, slice thicknesses, and using different gradient strengths. However, these variations have not affected ONSD measurement values, and the ONSD has been determined to range from 4.7 to 5.7 mm [7,9,14]. The normal value of the optic nerve sheath diameter (ONSD) is typically found to be between 4.4 and 5.4 mm on average [9]. In our study, we used a 1.5 Tesla MRI machine (Siemens Avanto, Germany), and we found the average optic nerve sheath diameter (ONSD) to be 4.87 mm.

The measurement of the optic nerve sheath diameter (ONSD) using MRI can indeed exhibit individual variations [6]. To minimize this variability, we conducted MRI measurements by appointing a consistent individual from our hospital’s competent radiology specialists. The positive correlations we found indicate the necessity of routinely reporting the ONSD in MRI evaluations.

Apache and expected death rate (EDR) are widely accepted important criteria for determining mortality. When we compared these criteria with ONSD measurement, we found significant correlation values. Thus, we supported our hypothesis that ONSD measurement helps determine mortality.

Cerebrovascular events (CVEs) are classified into two completely different categories: hemorrhagic and ischemic. Hemorrhagic CVEs are less common than ischemic CVEs [15]. Our case distribution also favored ischemic cerebrovascular events (CVEs). An important feature of our study is that we did not come across any study comparing ONSD measurements with hemorrhagic/ischemic CVE diagnoses. When we compared ONSD measurements with hemorrhagic/ischemic CVEs, we found a reverse correlation.

Research and prevention efforts are underway worldwide to investigate the etiology and prevention of cerebrovascular events (CVEs), which, following cardiovascular diseases, rank among the leading causes of disability and death globally. Disorders such as hyperglycemia and hyperlipidemia, along with comorbidities, increase the incidence of CVEs [2,16]. However, its impact on mortality is controversial [17]. When we compared the optic nerve sheath diameter (ONSD) with laboratory parameters such as kidney function tests, liver function tests, and glucose, we did not find a significant correlation. This is because the impact of these laboratory values on mortality is questionable, and these laboratory values do not have a direct effect on the optic nerve.

Upon examining hematological parameters obtained from complete blood counts, we found a significant correlation between the platelet count and ONSD. The role of inflammation in cerebrovascular events significantly influences clinical pathophysiology. Cerebral hypoxia, developing after ischemic stroke, disrupts energy metabolism at the cellular level, triggering neuroinflammatory processes. In the acute phase, the activation of immune cells occurs in both the central and peripheral nervous systems, with neutrophils, monocytes, and lymphocytes migrating to the ischemic or hemorrhagic lesion site, thereby increasing the release of proinflammatory cytokines [18]. During this process, the release of well-known proinflammatory cytokines such as TNF-α and IL-6 leads to the increased permeability of the blood–brain barrier (BBB) [19]. The inflammatory response occurring during cerebrovascular events is characterized by an increase in proinflammatory cytokines and reactive oxygen species (ROS). This increase enhances the permeability of cerebral vessels, potentially leading to the formation of vasogenic edema [20]. In our study, a positive correlation was observed between the optic nerve sheath diameter (ONSD) and white blood cell (WBC) count, suggesting that this relationship may be proportional to the severity of inflammation and that ONSD enlargement could serve as an indicator of the inflammatory process. Additionally, our study identified a positive correlation between the platelet count and ONSD. This finding suggests that platelets may be associated with thromboinflammatory processes in cerebrovascular events. In acute ischemic stroke, platelet activation may accelerate thrombosis formation in micro vessels, further impairing cerebral perfusion [21]. Moreover, platelets contribute to endothelial dysfunction by increasing the release of inflammatory mediators such as P-selectin and thromboxane A2. The findings indicate that ONSD measurements have the potential to reflect not only intracranial pressure changes but also the severity of systemic inflammation. The increase in WBC and platelet counts in acute stroke patients may be associated with inflammation and coagulation processes. Evaluating these parameters along with ONSD enlargement could provide valuable insights into clinical progression and prognosis.

Zhang et al. (2014) found a positive correlation in their study of 1556 patients investigating the relationship between thrombocyte indices and mortality in the intensive care unit [22]. There are very few publications on this topic. While measuring thrombocyte indices shows promise, its use in clinical practice has not yet become widespread [23]. Indeed, in our study, we did not find a significant correlation between the platelet count and mortality rate. According to our estimations, an increase in the platelet count is often associated with ischemic strokes, whereas our study also included hemorrhagic strokes.

We did not perform simultaneous invasive ICP measurements. However, since the patients were not under a follow-up protocol, we did not conduct invasive measurements. Of course, non-invasive tests are not sufficiently capable of replacing invasive ICP measurement. However, they can be used as an alternative in special cases, such as those with diagnoses like renal failure, meningitis, ischemic or hemorrhagic stroke, which do not require invasive monitoring [7]. Indeed, non-invasive tests offer advantages over invasive procedures for measuring ICP in patients with cerebrovascular events. These advantages include a lower risk of infection, no need for additional tests, and lower costs. However, measuring the ONSD with MRI may have some disadvantages, such as variability in measurements among individuals and the inability to be used in patients with ocular abnormalities [24]. Nevertheless, there are numerous positive outcomes reported in studies measuring the optic nerve diameter using imaging techniques [25].

Additionally, it is necessary to detail the longitudinal changes in ONSD thickness over time. Toscano et al. (2017) conducted a study using ONSD measurements during intensive care unit follow-ups and found that an increase in the ONSD of more than 0.7 mm is indicative of brain damage [26]. Similarly, Özdemir et al. (2022) found a strong positive correlation in their study, where they evaluated the prognosis of patients followed-up in the intensive care unit with serial measurements of the ONSD [27]. In our study, however, we examined the impact of patients’ initial presentation findings on prognosis. We found that as the ONSD increased, the sensitivity to mortality increased, as determined by ROC analysis.

Our study has some limitations. As a single-center retrospective study, it may be prone to biases related to data collection and generalizability. We did not account for all potential confounders, which may have influenced the results. Future multicenter studies with larger sample sizes and prospective designs are recommended.

## 5. Conclusions

Patients presenting to the emergency department with a suspected CVE undergo routine imaging techniques such as MRI for diagnosis and treatment purposes. Our study found a statistically significant positive correlation between the ONSD and mortality (r = 0.441, *p* = 0.002), as well as a significant association with expected mortality rate (r = 0.282, *p* = 0.004). Additionally, ROC analysis confirmed that increased ONSD values are associated with higher sensitivity in predicting mortality. These findings support the role of the ONSD as a non-invasive indicator of mortality. Alongside pathology investigation during MRI examination, we believe that ONSD measurement should be included in routine examination protocols. However, more comprehensive studies are needed for optimal measurement techniques.

## Figures and Tables

**Table 1:** Demographic characteristics.

Characteristic	n	Total
Female	62	102
Male	40	
Age Group		
18–45	9	102
46–65	32	
66+	61	

**Table 2:** Relationship between optic nerve sheath diameter and cranial region.

N = 102	Pearson Correlation Coefficient	*p*-Value
Brain Stem	−0.007	0.942
Frontal Lobe	−0.060	0.550
Skull Base	0.096	0.336
Temporal Lobe	0.127	0.204
Occipital Lobe	0.056	0.577
Cerebellum	0.102	0.305
Thalamus	−0.009	0.930
Right PCA	0.144	0.150
Brain Region	−0.016	0.875

N = number.

**Table 3:** Comparison of optic nerve sheath diameter with laboratory data.

N = 102	Pearson Correlation Coefficient	*p*-Value
Glucose	0.093	0.352
Urea	0.028	0.781
Creatinine	0.041	0.684
AST	0.033	0.740
ALT	0.003	0.979
ALP	0.027	0.786
GGT	0.126	0.206
Albumin	−0.065	0.516
Lipase	0.072	0.474
CK	−0.017	0.862
CRP	0.068	0.494

N: number, AST: Aspartate transaminase, ALT: Alanine transaminase, ALP: Alanine phosphatase, GGT: Gamma-glutamine transferase, CK: Creatine kinase, CRP: C-reactive protein.

**Table 4:** Comparison of optic nerve sheath diameter with laboratory data.

N = 102	Pearson Correlation Coefficient	*p*-Value
Ca	−0.165	0.098
PCT	0.108	0.279
P	0.007	0.942
Mg	−0.051	0.612
Na	−0.083	0.409
K	0.089	0.376
White Blood Cell	0.275	0.005
NEU %	0.081	0.420
LYM %	−0.122	0.220
MONO %	−0.045	0.652
HCT	0.049	0.622
PLT	0.200	0.044

N: number, Ca: Calcium, PCT: Procalcitonin, P: Phosphate, Mg: Magnesium, Na: Sodium, K: Potassium, NEU: Neutrophils, LYM: Lymphocytes, MONO: Monocytes, HCT: Hematocrit, PLT: Platelet.

**Table 5:** Sensitivity and specificity values for ROC analysis.

	Female		Male		Overall	
	(Sensitivity)	(Specificity)	(Sensitivity)	(Specificity)	(Sensitivity)	(Specificity)
Average Value − SD	0.268	0.952	0.222	1.000	0.294	0.941
Average Value	0.634	0.810	0.630	0.692	0.603	0.735
Average Value + SD	0.976	0.524	0.852	0.308	0.926	0.382

SD: Standard deviation.

## Data Availability

The datasets generated and analyzed during the current study are available from the corresponding author upon reasonable request.

## Abbreviations

The following abbreviations are used in this manuscript:

ONSD	Optic nerve sheath diameter
CVE	Cerebrovascular event
ICU	Intensive care unit
MRI	Magnetic resonance imaging
ICP	Intracranial pressure
APACHE II	Acute physiology and chronic health evolution II
GCS	Glasgow coma score
WBC	White blood cell
CRP	C-reactive protein
MPV	Mean platelet volume
EDR	Expected death rate
ROC	Receiving operator characteristic
AST	Aspartate transaminase
ALT	Alanine transaminase
ALP	Alkaline trans phosphatase
GGT	Gamma-glutamine transferase
CK	Creatine kinase
Na	Sodium
K	Potassium
Ca	Calcium
P	Phosphate
PCT	Procalcitonin
Mg	Magnesium
WBC	White blood cells
MONO	Monocytes
NEU	Neutrophils
LYM	Lymphocytes
HCT	Hematocrit
PLT	Platelet
